# Preliminary experience with the patient-specific templating total knee arthroplasty: 40 cases compared with a matched control group

**DOI:** 10.3109/17453674.2012.711700

**Published:** 2012-08-25

**Authors:** Bert Boonen, Martijn G M Schotanus, Nanne P Kort

**Affiliations:** Department of Orthopaedics, Orbis Medical Centre, Sittard-Geleen, The Netherlands

## Abstract

**Background and purpose:**

Patient-specific templating total knee arthroplasty (TKA) is a new method for alignment of a total knee arthroplasty that uses disposable guides. We present the results of the first 40 consecutive patients who were operated on using this technique.

**Methods:**

In this case-control study, we compared blood loss, operation time, and alignment of 40 TKAs performed using a patient-specific templating alignment technique with values from a matched control group of patients who were operated on by conventional intramedullary alignment technique. Alignment of the mechanical axis of the leg and flexion/extension and varus/valgus of the individual prosthesis components were measured on standing, long-leg, and standard lateral digital radiographs. The fraction of outliers (> 3˚) was determined.

**Results:**

Mean mechanical axis of templating TKAs was 181° with a fraction of outliers of 0.3, and mean mechanical axis of conventional TKAs was 179˚ (outlier fraction 0.5). Fraction of outliers in the frontal plane for femoral components was 0.05 in the templating TKAs and 0.4 in the conventional TKAs, and for tibial components the corresponding values were 0.2 and 0.2. In the templating TKAs and conventional TKAs, fraction of outliers in the sagittal plane was 0.4 and 0.9, respectively, for femoral components and 0.4 and 0.6 for tibial components. Mean operation time was 10 min shorter and blood loss was 60 mL less for templating TKA than for intramedullary-aligned TKAs.

**Interpretation:**

Patient-specific templating TKA showed improved accuracy of alignment and a small reduction in blood loss and operating time compared to intramedullary-aligned TKA, but the fraction of outliers was relatively high. Larger RCTs are needed for further evaluation of the technique and to define the future role of patient-specific template alignment techniques for TKA.

Nowadays, there are several methods for alignment of total knee arthroplasties (TKAs). These alignment methods can be divided into conventional techniques and navigational or image-guided surgery.

Complications associated with conventional techniques that use intramedullary alignment rods include extra blood loss perioperatively (Raut et al. 1993), embolization of medullary content (Caillouette and Anzel 1990, Fahmy et al. 1990), and difficulty in intramedullary rod passage due to deformity, retained hardware, or pathological bone disease (Dennis et al. 1993). An important factor influencing implant survival is the alignment of the mechanical axis; malalignment is associated with poorer survivorship (Lotke and Ecker 1977, Bargren et al. 1983, Jeffery et al. 1991, Ritter et al. 1994,), substantial change in pressure distribution (Hsu et al. 1990), and change in total load in the medial and lateral compartments of the tibial component (Werner et al. 2005). Computer navigation has been developed to improve implant and limb alignment and instability in conventionally placed prostheses (Beringer et al. 2007). A recent meta-analysis showed that malalignment of the mechanical axis of more than 3° occurs in one third of conventional TKA patients. In contrast, only one tenth of computer-assisted TKAs result in malalignment of the mechanical axis of more than 3° (Mason et al. 2007).

Peroperative navigation has some major drawbacks, however. They include the need for accurate landmark registration (Lombardi et al. 2008), increased surgical time and cost (Radermacher et al. 1998, Lombardi et al. 2008), pin loosening and bone fractures (Lombardi et al. 2008), complexity (Radermacher et al. 1998), long set-up time (Radermacher et al. 1998), and a substantial learning curve (Lombardi et al. 2008).

Recently, a patient-specific alignment guide, Signature Personalized Patient Care (SPPC) (Biomet Inc., Warsaw, IN) was developed, based on a preoperative MRI scan of the patient’s leg. With this alignment guide the intramedullary cavity is not opened, thus eliminating the risks associated with it. In addition, the new technique theoretically eliminates most of the disadvantages of intraoperative navigation.

We present the preliminary results of our first 40 consecutive cases operated with this new technique and compared them with results from a matched control group operated using conventional intramedullary alignment technique. We expected operation time and degree of blood loss to be lower in the SPPC group. Alignment, in terms of fraction of outliers, was expected to be superior in the SPPC group than in the conventional intramedullary alignment technique.

## Patients and methods

40 knees in 39 patients (25 women) with osteoarthritis were operated on by means of the SPPC procedure between December 2009 and March 2010, and were eligible for inclusion in this case-control study. We excluded patients with a BMI above 35, patients with a history of osteotomy, and patients with metal near the knee joint.

Preoperative MRI scanning of the hip, knee, and ankle was performed 6 weeks before surgery according to a standard scanning protocol to determine the mechanical axis of the leg.

Software (Materialise NV, Leuven, Belgium) was used to create virtual 3D models of the femur and tibia. Then the program was used to determine appropriate implant size and optimal positioning of the prosthesis (Vanguard Complete Knee System; Biomet Inc., Warsaw, IN). Component sizing was determined by measuring the AP dimension of the distal femur and the contour of the proximal tibia. Planned implant size was the best fitting size of a range of 10 standard femoral and tibial components of the Vanguard Knee System. Position of the prosthesis was calculated to obtain a neutral mechanical axis and a neutral position of femoral and tibial components relative to the mechanical axes of femur and tibia in the frontal plane. In the sagittal plane, posterior slope of the tibial component and flexion of the femoral component were set at 3 degrees. Femoral rotation was set parallel to the transepicondylar axis in the coronal plane. Rotation of the tibial component could not be calculated preoperatively using software. A digital, virtual plan of the operation to be performed was sent to the surgeon. For the femoral side, the plan showed the templated size; anteroposterior (AP), mediolateral, and bottom views with and without the implant; a visual angle overview of the femur; summary tables of the angles and levels of resection; and visuals showing areas of overhang. For the tibial side, the plan showed visuals of AP, mediolateral, and top views of the tibia with planned resection and summary tables of the angle and level of resection. The surgeon was able to adjust the digital plan by changing implant size and position (rotation and translation), inclination/posterior slope, and resection level. However, we did not make any changes to this proposed plan and we only approved the calculations provided by the software.

**Figure 1.**
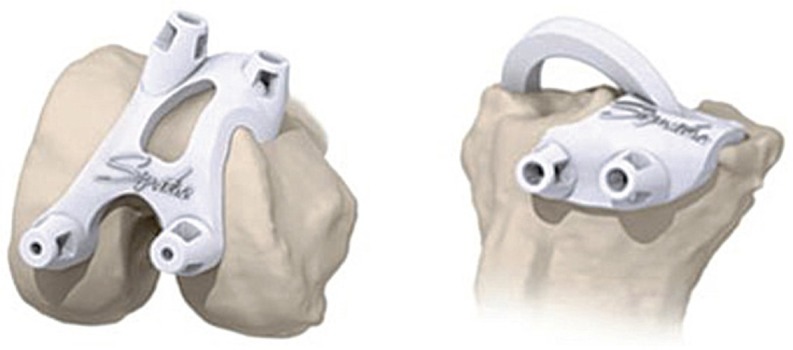
SPPC alignment guides for femur and tibia.

The patient-specific disposable guides made of polyamide (Figure 1) were then manufactured.

SPPC guides and an overview of the surgical plan were delivered to our hospital 2–3 days before surgery.

**Figure 2.**
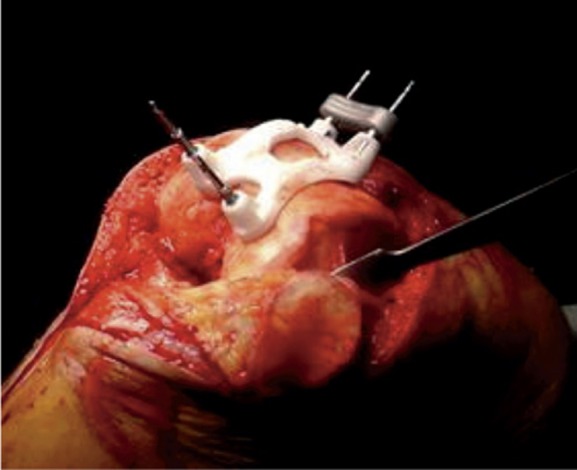
Placement of guide on femur.

**Figure 3.**
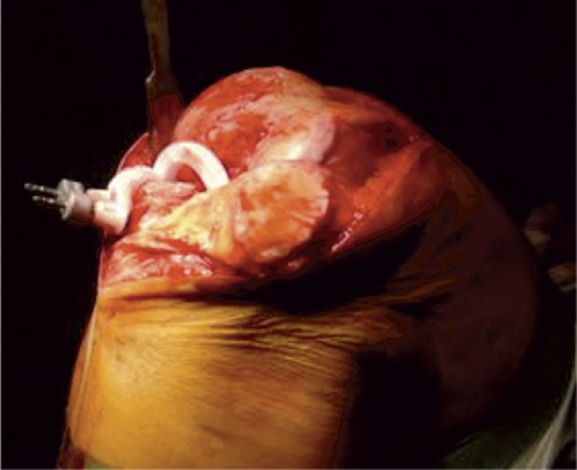
Placement of guide on tibia.

In all patients, a standard midline incision with a medial parapatellar arthrotomy was performed and standard exposure of the femur and tibia was carried out with patella eversion. The anterior cruciate ligament was sacrificed and the posterior cruciate ligament was preserved in all patients. The patient-specific guides were placed on the femur and tibia (Figures 2 and 3) in the fitting position with the osteophytes still in place; calculations for the fitting of the guides are made considering these osteophytes. Furthermore, we paid attention to whether or not there was a mismatch between the guides and the articular surface of the knee. When there was mismatch, this was registered on the operative record form.

The femoral guide allowed the surgeon to place pins for both the distal cutting block and the 4-in-1 cutting block to make distal, anterior, posterior, and chamfer cuts. The tibial guide was used to place pins for the horizontal cut block of the knee system. After pin placement, levels of resection could be adjusted in steps of 2 mm and the size of the femoral component could be changed—as this mechanism is incorporated in the distal and horizontal cut blocks of the standard Vanguard Knee System. The bone cuts were made using traditional saw blades. A trial femoral and tibial component of the prosthesis was inserted to check whether the positioning was adequate. Rotational alignment of the tibial component was performed using an extramedullary rod, which was pointed toward the second metatarsal bone. Drill holes corresponding to design of the definite prosthesis were made in the distal femur and proximal tibia using these trial components.

Blood loss was registered at this point and a tourniquet was inflated to 350 mmHg prior to extensively rinsing the knee with a pulse-lavage system. A total knee system was placed. The patellae were resurfaced in 3 cases. Soft tissue releases, medially or laterally, were performed if necessary. A Bellovac ABT drain (Astratech, Mölndal, Sweden) was placed and the arthrotomy was closed in layers.

An operative record was completed, containing information on operation time (min from incision until the bandage was placed), blood loss (mL of blood in the suction device, prior to application of a tourniquet and prior to rinsing the knee), size of the components and polyethylene insert. Blood loss and operation time were recorded by an independent OR nurse.

Femoral nerve block was used in all patients, and the catheter was removed on the second day after surgery. Oral analgesics were administered according to the standard pain protocol. Arixtra (5 mg/mL, 0.5 mL; GlaxoSmithKline) was used as thrombo-embolic prophylaxis for 5 weeks after surgery. All patients participated in a rapid recovery program (Joint Care; Biomet) The criteria for discharge of patients were: dry wound, flexion of the knee up to at least 90°, and the ability to climb stairs.

40 patients who had been operated on by the same surgeon between April 2008 and July 2009 using the conventional technique were matched to the SPPC group. The matching was done on the following variables: type of implant (Vanguard), patient treated in rapid recovery program, sex, and age. Matching was done by searching the operative record used in our hospital. The operative procedure was identical to that in SPPC patients, except for pin placement for the cut blocks, which was done using standard intramedullar alignment guides. Femoral components were placed in 3° valgus relative to the anatomical axis of the femur. Tibial components were placed perpendicular to the anatomical axis of the tibia. Femoral component flexion and tibial posterior slope were set at 0°. The operative record that was completed was the same as in the SPPC group. In addition, BMI was calculated for selected patients to check for relevant differences between the two groups.

Mean operation time and mean blood loss in the SPPC group were compared to corresponding values in the matched control group. Data were obtained from the operative record.

Default sizes of the femoral and tibial components and polyethylene insert as calculated with software prior to surgery were compared to the actual sizes of the femoral and tibial components and the polyethylene thickness in the SPPC group.

**Figure 4:**
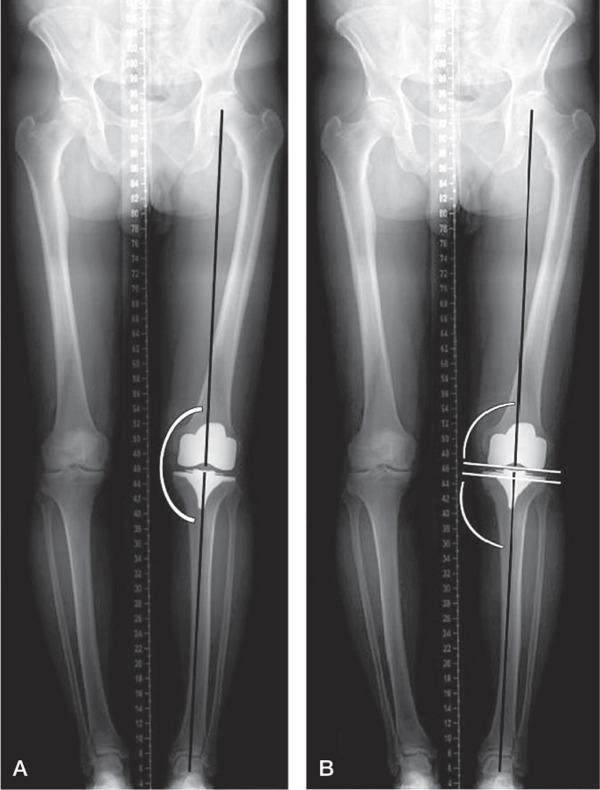
A. long-leg radiograph. Mechanical axis of the leg measured on a standing long-leg radiograph as the medial angle between the mechanical axis of the femur, defined as the line between the center of the hip and the center of the femoral component, and the mechanical axis of the tibia, defined as the line between the center of the tibial component and the center of the ankle. B. Long-leg radiograph. FFC and FTC angles were measured as the medial angles between the femoral component and the mechanical axis of the femur and between the tibial component and the mechanical axis of the tibia, respectively.

Standing, weight-bearing, AP long-leg digital radiographs were taken preoperatively and 6 weeks postoperatively in the SPPC group. We asked the patients in the control group to return to the hospital for long-leg digital radiographs. Standard lateral radiographs were already available for this control group. The mechanical axis was determined according to Tigani et al. (2009) (Figure 4A) and was measured using calibrated software. Deviations of more than 3° from a neutral mechanical axis were regarded as outliers, and fractions were calculated.

The varus/valgus position of the femoral and tibial components (frontal femoral component (FFC) angle and frontal tibial component (FTC) angle), relative to the mechanical axis, was measured on the same long-leg radiographs (Figure 4B). Values of more than 90° indicate valgus positioning of the femoral and tibial component and values less than 90° indicate varus positioning. Fractions of outliers with more than 3° varus or valgus were calculated.

**Figure 5.**
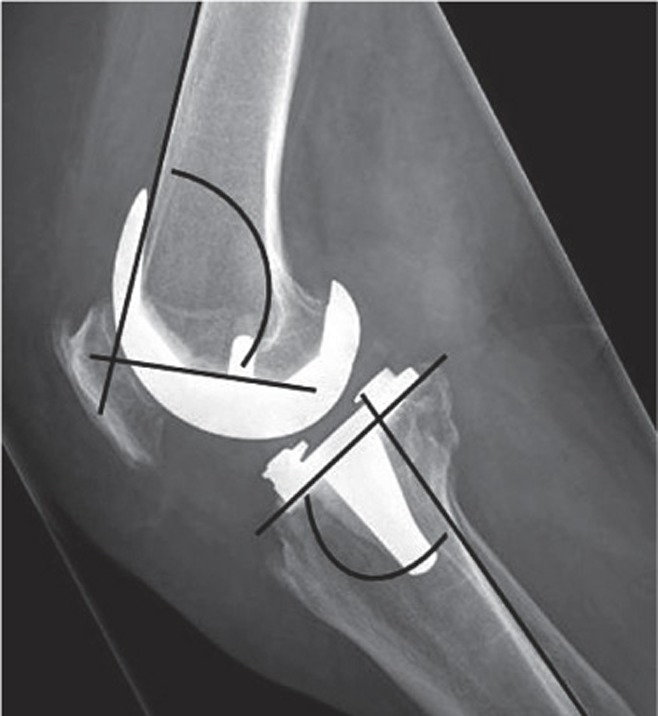
Lateral radiograph showing the LFC and LTC angles. LFC angle was defined as the anterior angle between the femoral component and the cortex of the femur. LTC angle was defined as the angle between the tibial component and the posterior cortex of the tibia.

Femoral component flexion and tibial component posterior slope (lateral femoral component (LFC) angle and lateral tibial component (LTC) angle, respectively) were measured on standard lateral radiographs according to Tigani et al. (2009) (Figure 5). Fractions of outliers of more than 3° were calculated. Lateral radiographs were taken 6 weeks postoperatively with the knee in a slight degree of flexion.

All radiographic measurements were performed by an independent reviewer who was blind regarding the treatment groups. 3 measurements were conducted separately to obtain intraobserver reliability by calculating the intraclass correlation coefficient.

### Statistics

Student’s t-test was used to compare blood loss, operation time, and alignment of mechanical axis and individual femoral and tibial components between the SPPC group and the matched control group. Mann-Whitney test was used to compare fractions of outliers between groups. Values of p < 0.05 were considered significant. We used SPSS software.

## Results

Patients in the SPPC group (n = 40) were adequately matched to a control group (n = 40) with respect to age, sex, and operative procedure. Mean age was 68 years in both groups, and there were 25 women in each group. Mean BMI indices for both groups indicated an overall overweight (non-obese) classification of patients. Mean preoperative mechanical axis in the SPPC group was 175° (range 162–188, SD 6.4). 31 of 40 had varus mechanical axis (range 162–178°) and 9 of the 40 had valgus mechanical axis (range 180–188°). No preoperative long-leg radiographs were taken in the control group.

### Operative data

Mean operation time and mean blood loss were statistically significantly lower in the SPPC group. Femoral, tibial, and insert size were similar in both groups (Table 1).

**Table 1.** Prosthesis and OR data. Mean (SD)

	SPPC	Conventional	p-value
Femoral size	66 (4)	65 (4)	0.3
Tibial size	74 (5)	74 (5)	0.5
Insert thickness	11 (1)	12 (2)	0.2
OR time, min	51 (11)	61 (14)	0.001
Blood loss, mL	239 (95)	299 (115)	0.01

Actual femoral component size, tibial size, and insert thickness were all statistically significantly different from the default size and thickness in the SPPC group (Table 2). In 8 cases, resections were altered peroperatively for the femoral component and in 10 cases they were altered for the tibial component. Insert thickness was changed from standard (10 mm) to 12 mm in 16 cases and to 14 mm in 6 cases. The individual guides fitted well on the native bone and cartilage.

**Table 2.** Default setting and OR data. Mean (SD)

	Default	OR data	p-value
Femoral size	67 (5)	66 (4)	0.01
Tibial size	72 (6)	74 (5)	0.001
Insert thickness	10 (0)	11 (1)	< 0.001

### Radiographic evaluation

*Mechanical axis.* In 2 radiographs in the SPPC group, measurements could not be performed. The ankle joint was not visible and the mechanical axis could therefore not be determined. 35 patients in the conventional group returned for long-leg radiographs.

Mean values of mechanical axis, range, and number of outliers were compared between the groups and intraclass correlation coefficients were obtained for all measurements (Table 3). Fraction of outliers was not statistically significantly different in either group: 0.3 in the SPPC group and 0.5 in the conventional group.

**Table 3.** Mechanical axis

	SPPC (n = 38)	Conventional (n = 35)	p-value
Mean (SD)	181° (4)	179° (3)	0.02
Range	171–188°	175–185°	
Number of outliers > 3°	11/38	16/35	0.1
ICC	0.99	0.99	

*FFC angle and FTC angle.* In 2 radiographs in the SPPC group, measurements could not be performed because the ankle joint was not visible. In 1 radiograph from the conventional group, measurements were impossible because of over-projection of the femoral and tibial components. Mean values, range, and number of outliers were calculated and compared between groups (Table 4). There was a statistically significant difference in fraction of outliers for FFC angle in favor of the SPPC group. No such significant difference could be found for the FTC angle.

**Table 4.** FFC and FTC

	SPPC (n = 38)	Conventional (n = 34)	p-value
FFC, mean (SD)	90° (2)	88° (2)	< 0.001
FFC, range	84– 93°	85–92°	
FFC, number of outliers > 3°	2/38	12/34	0.001
FFC, ICC	0.99	0.82	
			
FTC, mean (SD)	91° (2)	91° (2)	0.7
FTC, range	87–96°	87–95°	
FTC, number of outliers > 3°	7/38	7/34	0.8
FTC, ICC	0.99	0.99	

*LFC angle and LTC angle.* One radiograph in the SPPC group and 2 in the conventional group were of unacceptable quality for performance of measurements. Mean values, range, and number of outliers were calculated and compared between groups (Table 5). There was a statistically significant difference in fraction of outliers for both the LFC angle and the LTC angle, both in favor of the SPPC group.

**Table 5.** LFC and LTC

	SPPC (n = 39)	Conventional (n = 38)	p-value
LFC, mean (SD)	85° (4)	84° (3)	0.1
LFC, range	74–94°	79–89°	
LFC, number of outliers > 3°	16/39	33/38	< 0.001
LFC, ICC	0.99	0.99	
			
LTC, mean (SD)	94° (4)	87° (3)	< 0.001
LTC, range	87–102°	75–92°	
LTC, number of outliers > 3°	14	21	0.02
LTC, ICC	0.99	0.99	

## Discussion

### Alignment in the frontal plane

Optimal alignment in the frontal plane has generally been considered to be within 3˚ varus/valgus of the mechanical axis (Lotke and Ecker 1977, Jeffery et al. 1991, Ritter et al. 1994, Archibeck and White 2002). More recently, however, it has been hypothesized that the 3° range for optimal alignment is an arbitrary figure, and that it is more likely that any deviation from neutral will reduce longevity by an amount that is proportional to the malalignment (Sikorski 2008). Furthermore, a distinction has to be made between restoration of a neutral mechanical axis and the optimal position of the individual components relative to this mechanical axis. Ideally, the position of the femoral and tibial components is perpendicular to the mechanical axis of the femur and tibia, respectively (Sikorski 2008).

Mean mechanical axis in our SPPC series was 181° (± 4°). We observed outliers of more than 3˚ varus/valgus in 12 of 40 patients. The fraction of outliers was not statistically significantly lower in the SPPC group than in the conventional group. This was not what we had expected. Several explanations can be given for this observation. This cohort consisted of the first 40 consecutive patients who were operated on with this new technique, and it is therefore likely that there was a learning curve. Particularly for the tibial component, it takes a number of cases before a surgeon learns how to remove soft tissues and how to adequately position the guide on the native bone. However, the outliers were more or less evenly distributed among the SPPC cohort. In itself, a learning curve would therefore be an insufficient explanation. We hypothesize that the higher than expected fraction is probably due—at least in part—to the fact that 5 patients were unable to fully extend the knee when long-leg radiographs were taken. This may result in possible inaccuracies in measurement (Krackow et al. 1990, Jeffery et al. 1991, Swanson et al. 2000, Hauschild et al. 2010).

In a meta-analysis, Mason et al. reported results of mechanical axis alignment outcomes for navigated and conventional techniques in TKA (Mason et al. 2007). A malalignment of the mechanical axis of greater than 3° occurred in 9% of patients in the navigated TKA group, as opposed to 32% of patients in the conventional group. We expected our results to be comparable to peroperatively navigated TKA, but the fractions of outliers differed substantially from the results reported by Mason et al.

For individual femoral and tibial components, there was a higher proportion of malalignment in the conventional group than in the SPPC group. Mason et al. (2007) reported similar observations when comparing peroperatively navigated TKA with conventional TKA. Malalignment of the femoral component was observed in 5% of navigated TKAs and tibial component malalignment was observed in 4%. We expected our fraction of outliers to be comparable to that in peroperatively navigated TKA, but there was a higher fraction—mainly for the tibial component. As stated before, we believe that this was mainly the result of the learning curve. However, given these results, low accuracy of the planning software must also be considered as one of the causes of malalignment.

### Alignment in the sagittal plane

We calculated the outliers in alignment considering both an ideal femoral component flexion and a tibial component posterior slope of 3° in the SPPC group. In the conventional group, the outliers were calculated considering an ideal femoral component flexion and tibial component posterior slope of 0°, as the intramedullary system is designed to give this alignment.

The fractions of outliers of the femoral and tibial components of more than 3° were statistically significantly lower for the SPPC group than for the conventional group.

We observed an overall high fraction of outliers in the sagittal plane. As hypothesized by others, this could be the result of the high variability of the femoral cortex (Tigani et al. 2009). However, this combined with our observation of a higher than expected fraction of outliers in the mechanical axis and in the position of the tibial component in the frontal plane in our series means that we must again raise the question of accuracy of the software and production process to create the SPPC guides. The producer of the guides claims that there is an extremely high level of accuracy, however. We have set up a study to further compare postoperatively achieved alignment with the alignment in the digital plan as calculated with software. Measurements will be performed using CT-scan, as this is considered to be the most accurate method for measurement of lower limb alignment (Chauhan et al. 2004).

Also of interest is our observation that default femoral and tibial size and insert thickness differed from actually placed sizes and thicknesses. The software calculates resections for placement of a 10-mm insert and we did not make changes to these calculations preoperatively. The difference in calculated and placed polyethylene thickness can be explained by the fact that in some cases, the resection level was changed intraoperatively. The software calculates very conservative bone cuts, and in some cases this would have resulted in a resection level through very sclerotic bone with a higher chance of malalignment due to deviation of the sawing blade. In these cases, adjustments were made for an extra resection with an accompanying need for thicker PE insert. Unfortunately, we were unable to obtain data on the exact number of cases in which adjustments were made peroperatively. We aim to address this issue further in the study that is currently being set up.

Also, for the sizing of the components, we did rely on the preoperative plan without making changes to it. Difference in size of the femoral component is probably due to the fact that the software overestimates the size because calculations try to avoid notching of the femoral component in any case. For the tibial component, the software tries to avoid overhang at all times, and this is most likely the explanation of why a larger tibial component could be placed than was calculated by software.

Although adjustments sometimes had to be made intraoperatively for the reasons given above, we had to use less soft tissue balancing techniques in the SPPC group than in the conventional group. Only in extreme varus or valgus deformities were soft tissue releases deemed necessary. It is important to consistently check the digital preoperative plan and to make adjustments to it where appropriate.

### Blood loss and operation time

Blood loss was 60 mL less in the SPPC group. The difference is most probably due to not having to open the intramedullary canal of the femur and tibia and the shorter operation time in the SPPC group.

The difference in operation time was 10 min because of the fewer surgical steps needed to implant the knee. In our experience, additional time can be saved when one also considers the time needed to install instruments on sterile fields, because fewer instruments are needed to perform the operation.

### Limitations and strengths of the study

The limitations of the present study were the relatively low number of patients included and the alignment measures that were performed by only 1 independent reviewer, with the known pitfalls and inaccuracies of measurements performed on standing long-leg radiographs. Restrictions of retrospective data analysis naturally apply.

The strength of our study was the direct comparison of outcomes in the SPPC group with those in a recent well-matched control group, operated on by the same surgeon.

Future research should focus on investigating (1) alignment with more reliable techniques (CT-scan) to make sure that the surgeon can rely on the digital preoperative plan, (2) the clinical outcome of the system, and (3) additional expenses and costs saved with the procedure. Larger randomized controlled trials to compare conventional intramedullary alignment with preoperative navigation in TKA are therefore needed.
